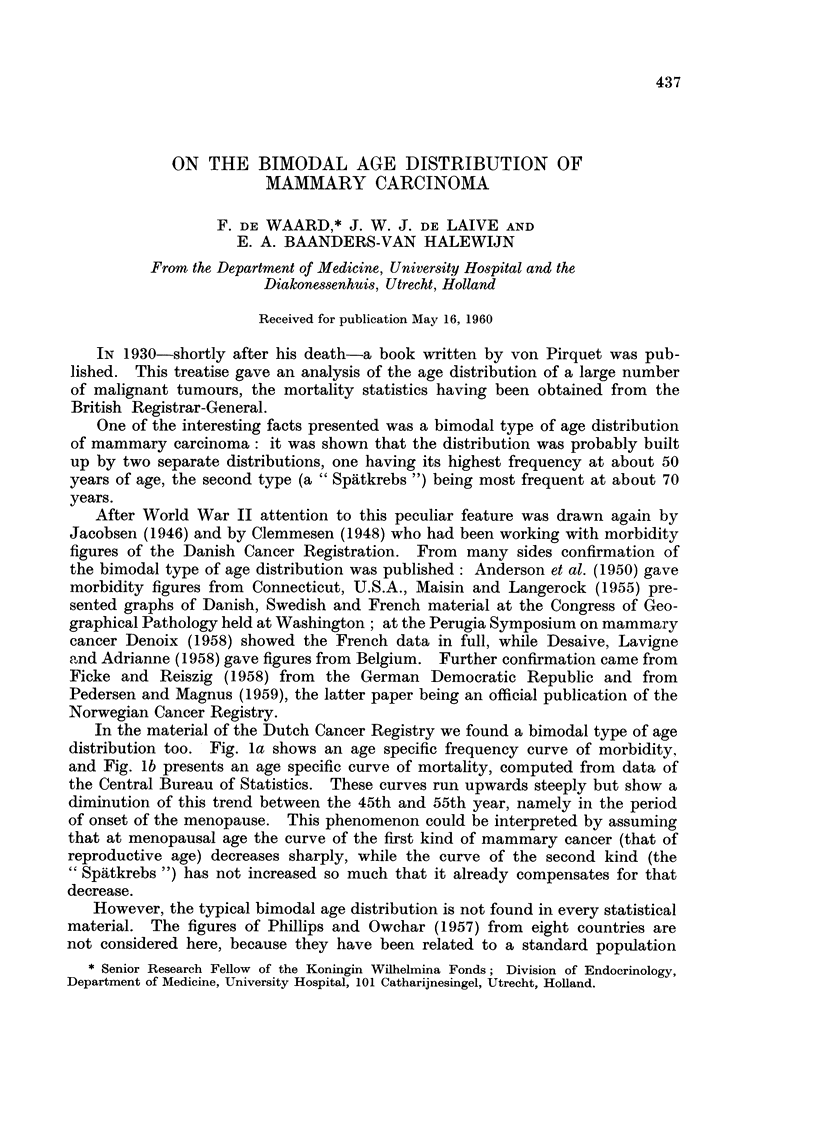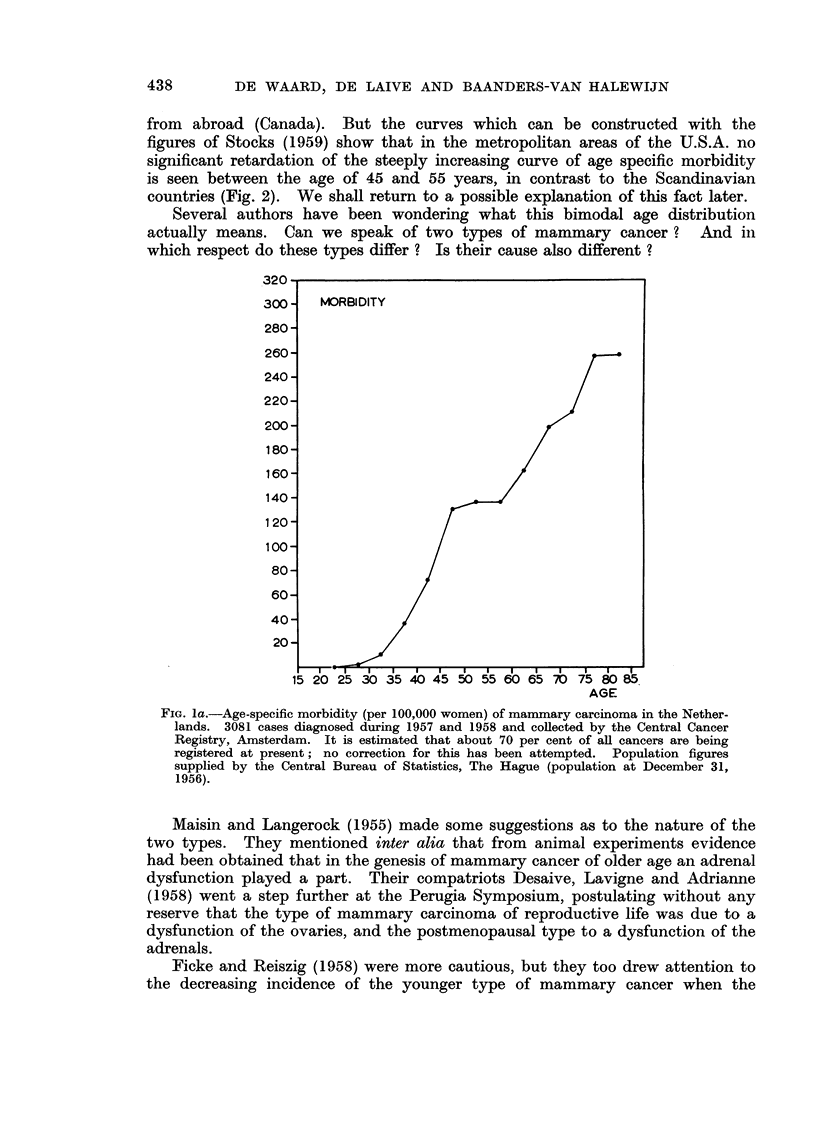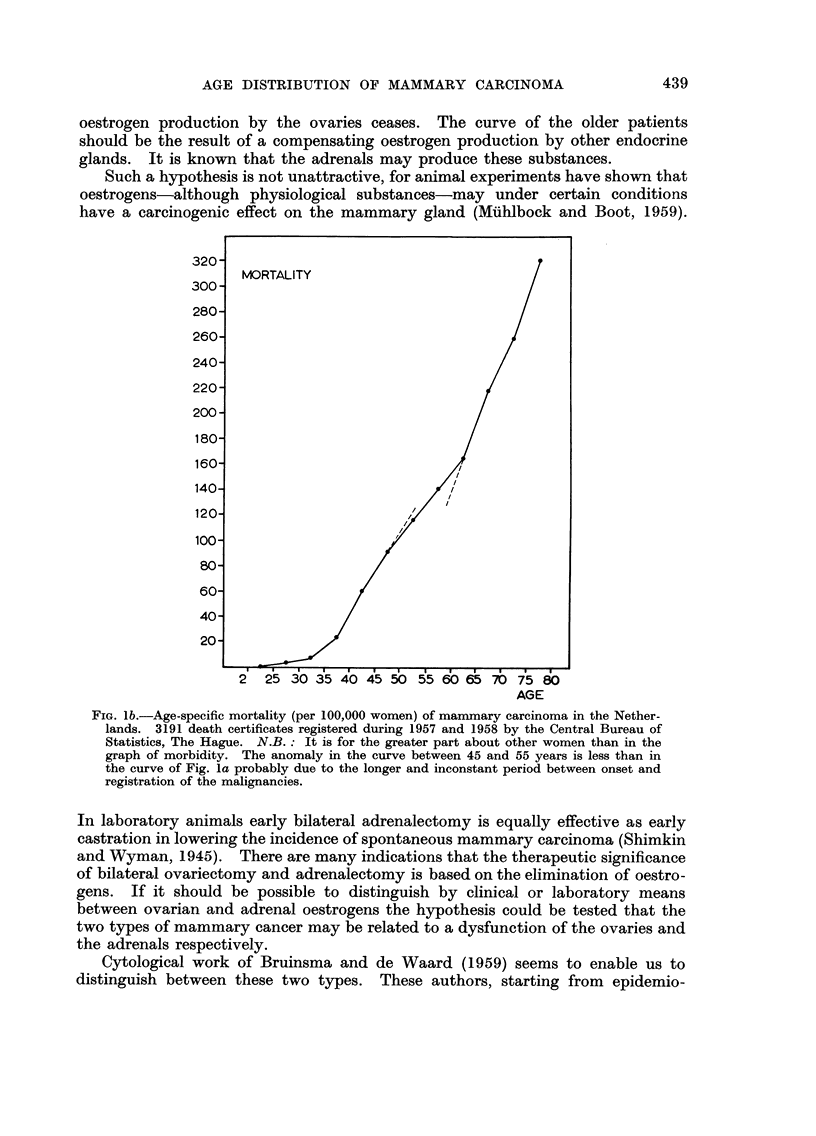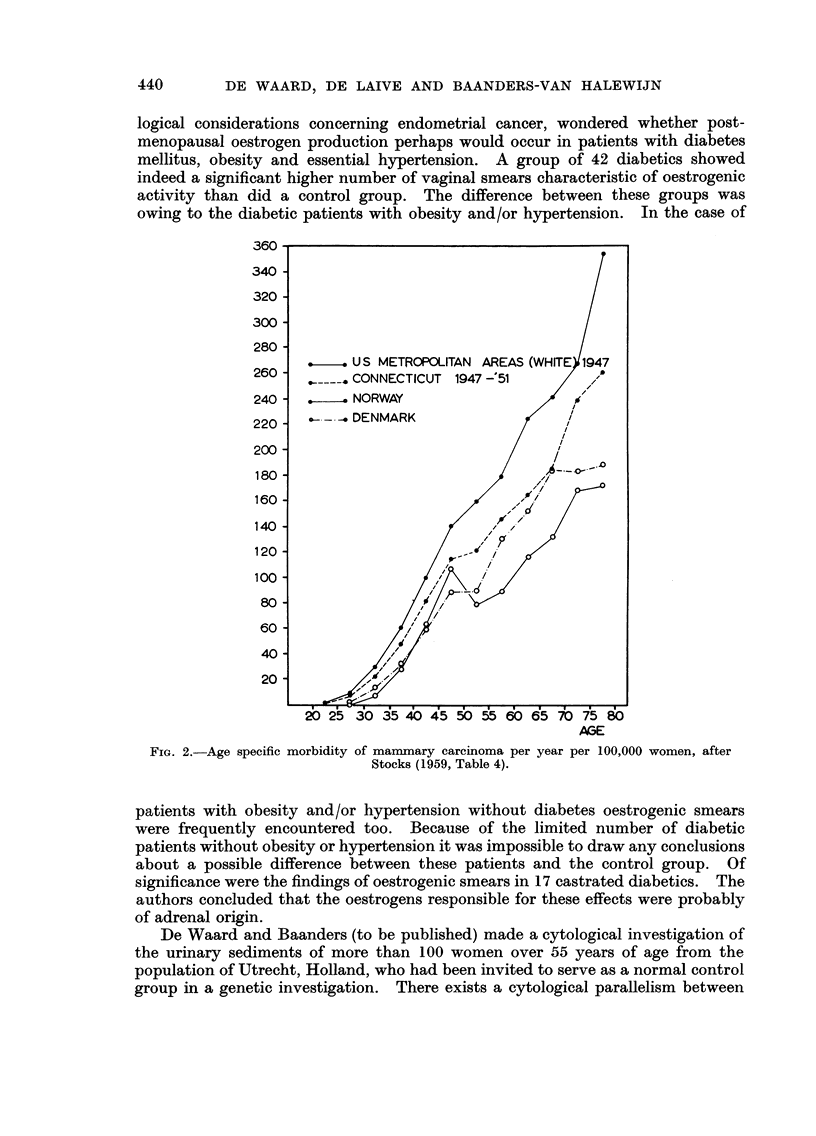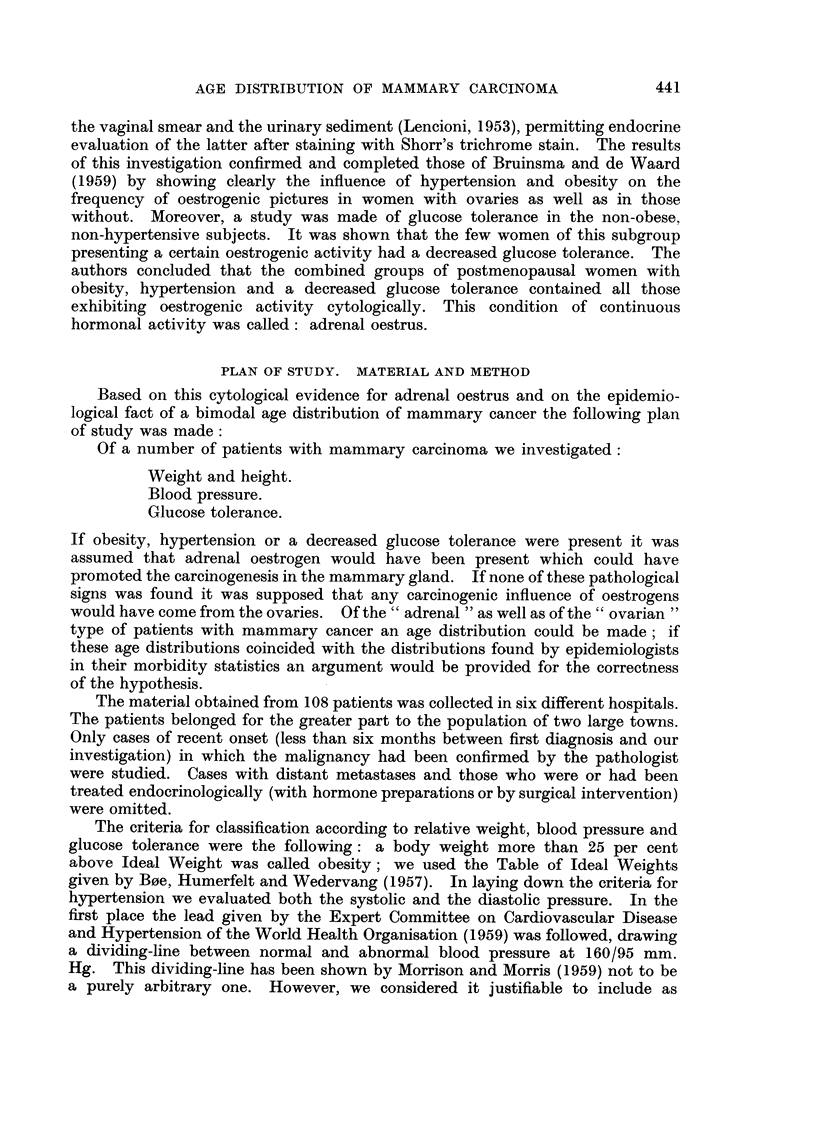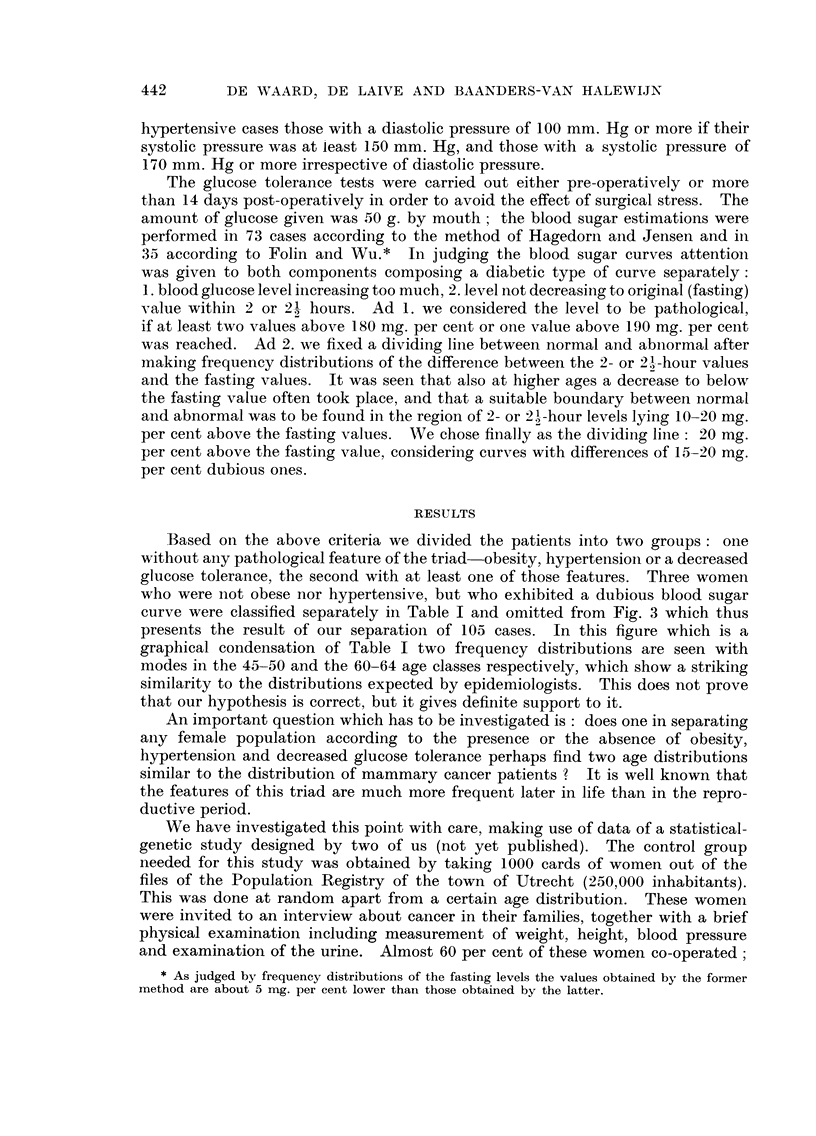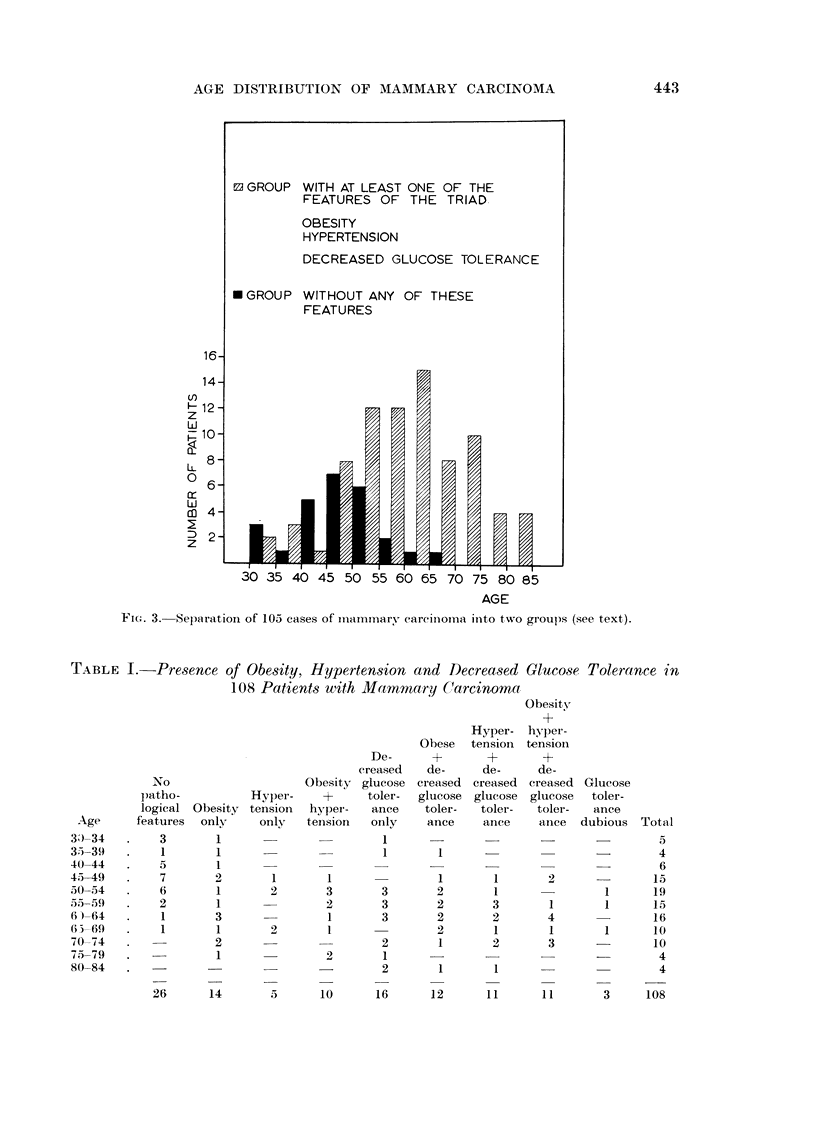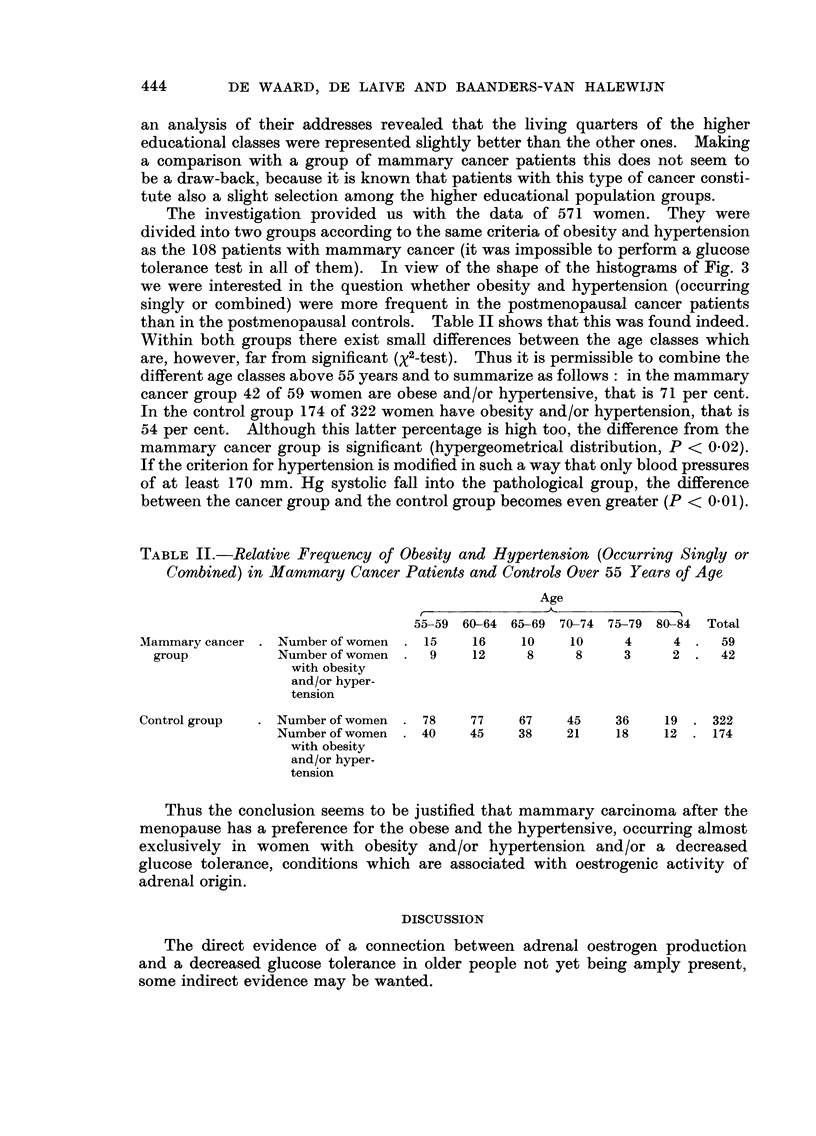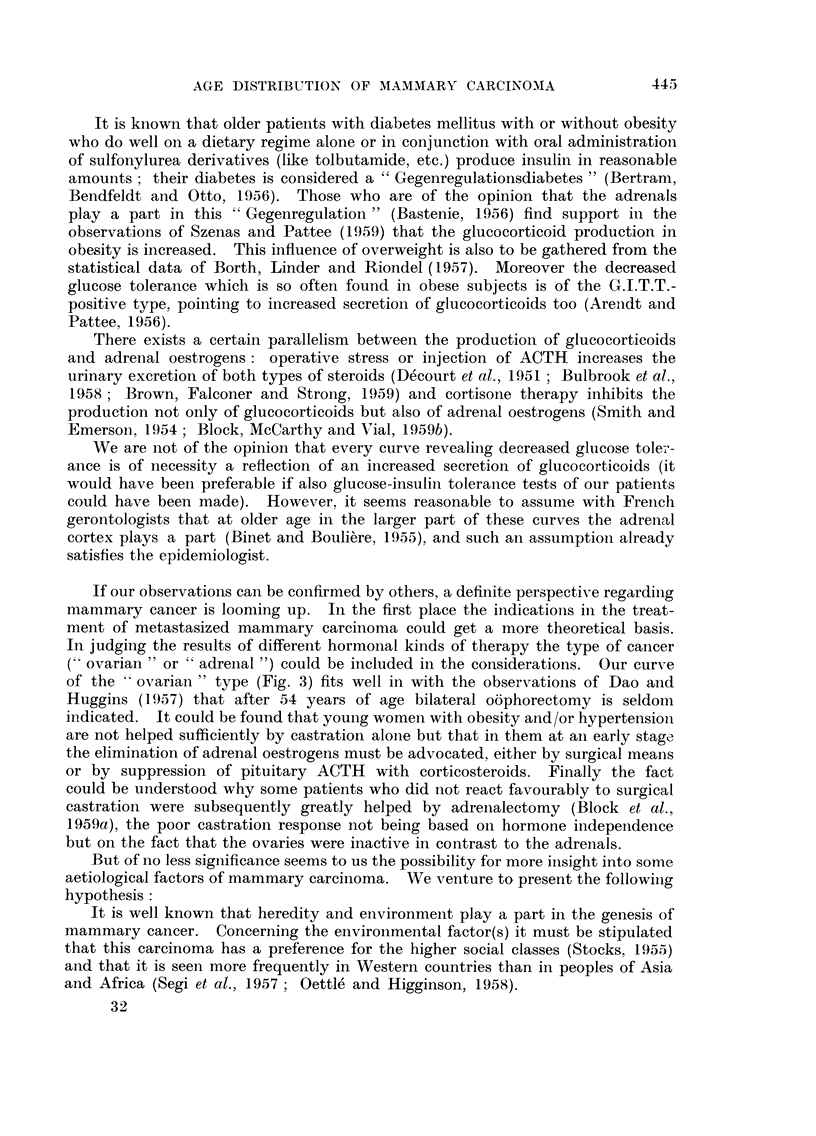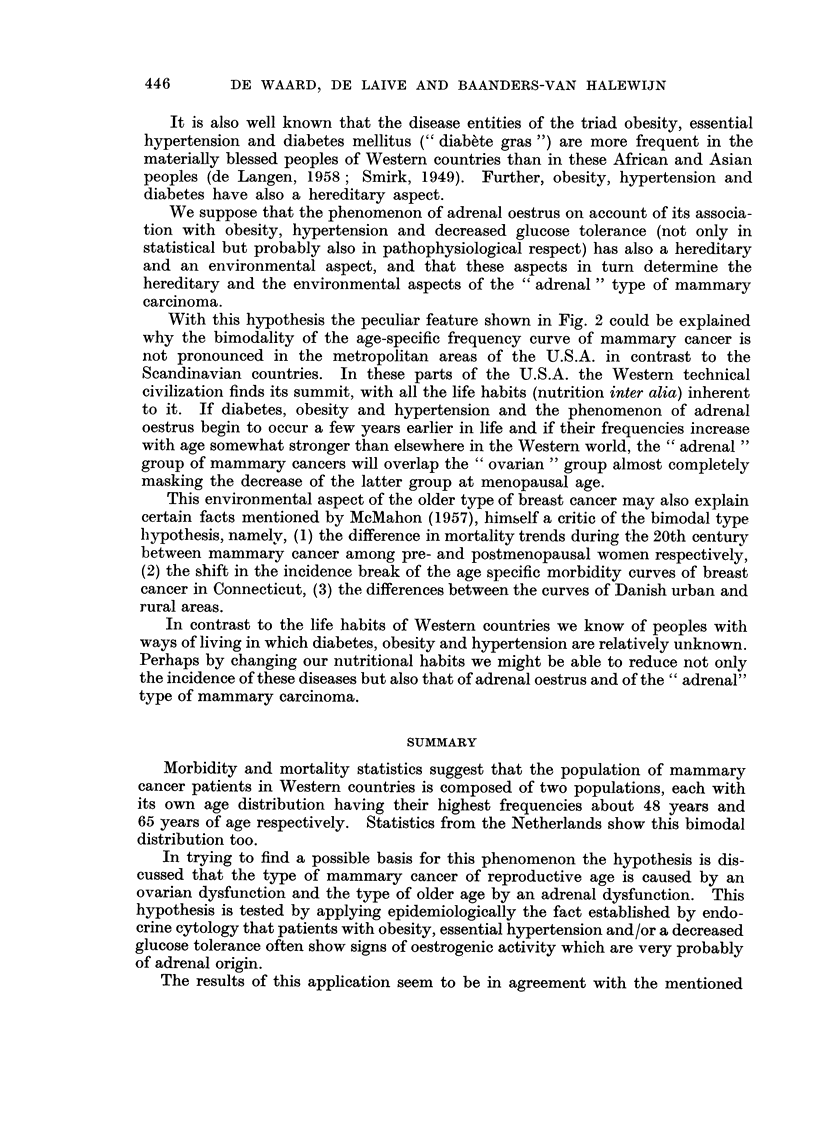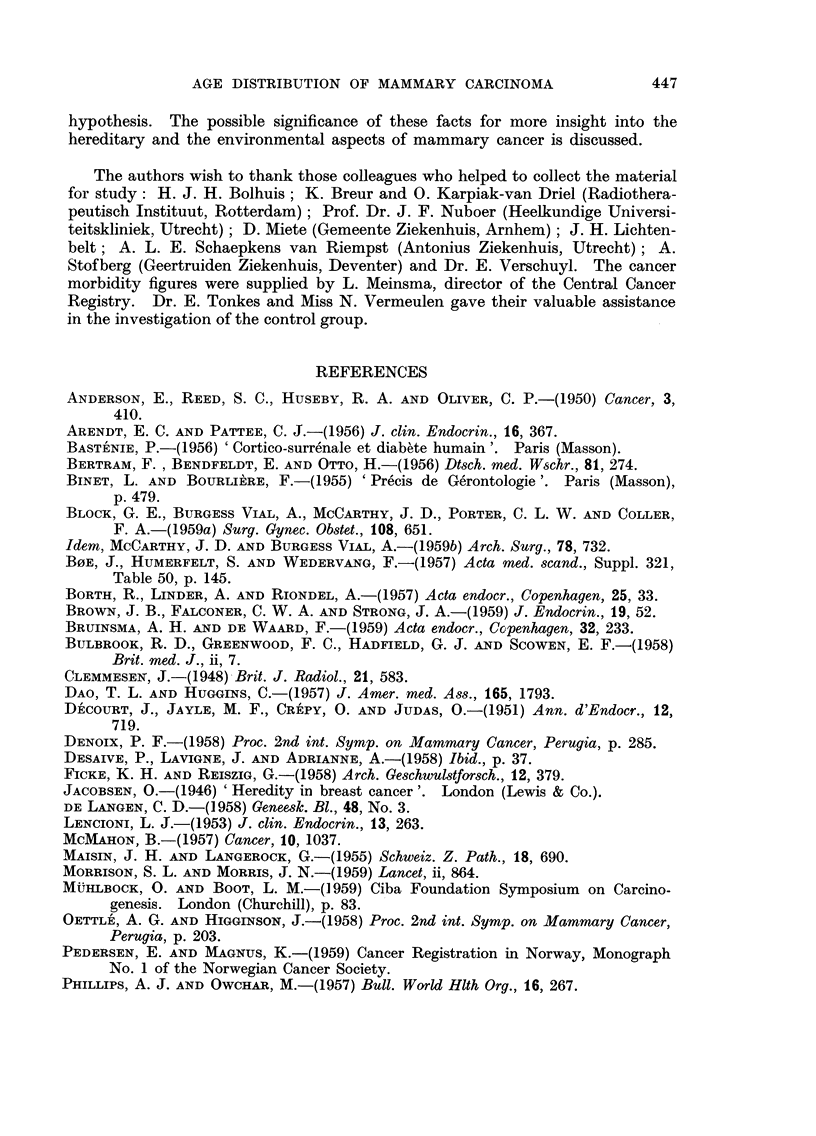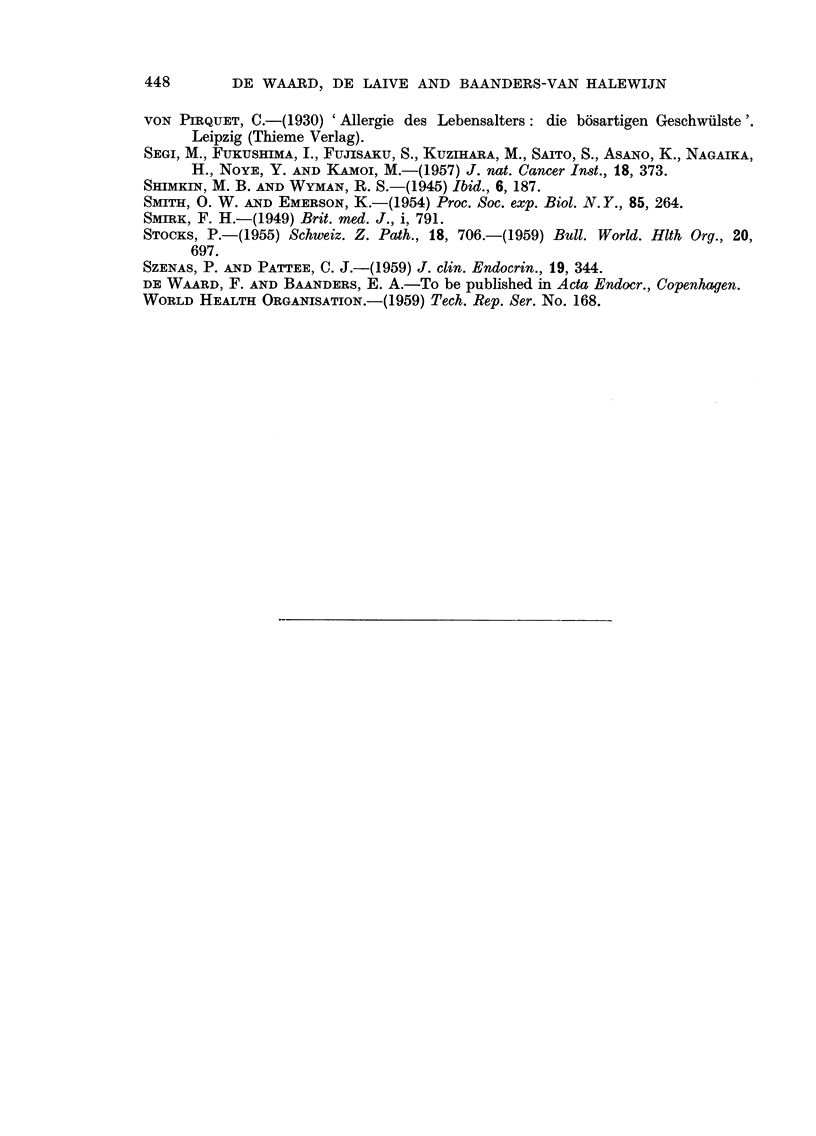# On the Bimodal Age Distribution of Mammary Carcinoma

**DOI:** 10.1038/bjc.1960.47

**Published:** 1960-09

**Authors:** F. de Waard, J. W. J. de Laive, E. A. Baanders-van Halewijn


### ON THE BIMODAL AGE DISTRIBUTION OF MAMMARY CARCINOMA

F. DE WAARD,* J. W. J. DE LAIVE AND
E. A. BAANDERS-VAN HALEWIJN

*From the Department of Medicine, University Hospital and the Diakonessenhuis, Utrecht, Holland*

Received for publication May 16, 1960

IN 1930—shortly after his death—a book written by von Pirquet was published. This treatise gave an analysis of the age distribution of a large number of malignant tumours, the mortality statistics having been obtained from the British Registrar-General.

One of the interesting facts presented was a bimodal type of age distribution of mammary carcinoma: it was shown that the distribution was probably built up by two separate distributions, one having its highest frequency at about 50 years of age, the second type (a " Spätkrebs ") being most frequent at about 70 years.

After World War II attention to this peculiar feature was drawn again by Jacobsen (1946) and by Clemmesen (1948) who had been working with morbidity figures of the Danish Cancer Registration. From many sides confirmation of the bimodal type of age distribution was published: Anderson *et al.* (1950) gave morbidity figures from Connecticut, U.S.A., Maisin and Langerock (1955) presented graphs of Danish, Swedish and French material at the Congress of Geographical Pathology held at Washington; at the Perugia Symposium on mammary cancer Denoix (1958) showed the French data in full, while Desaive, Lavigne and Adrianne (1958) gave figures from Belgium. Further confirmation came from Ficke and Reiszig (1958) from the German Democratic Republic and from Pedersen and Magnus (1959), the latter paper being an official publication of the Norwegian Cancer Registry.

In the material of the Dutch Cancer Registry we found a bimodal type of age distribution too. Fig. 1*a* shows an age specific frequency curve of morbidity, and Fig. 1*b* presents an age specific curve of mortality, computed from data of the Central Bureau of Statistics. These curves run upwards steeply but show a diminution of this trend between the 45th and 55th year, namely in the period of onset of the menopause. This phenomenon could be interpreted by assuming that at menopausal age the curve of the first kind of mammary cancer (that of reproductive age) decreases sharply, while the curve of the second kind (the " Spätkrebs ") has not increased so much that it already compensates for that decrease.

However, the typical bimodal age distribution is not found in every statistical material. The figures of Phillips and Owchar (1957) from eight countries are not considered here, because they have been related to a standard population

* Senior Research Fellow of the Koningin Wilhelmina Fonds; Division of Endocrinology, Department of Medicine, University Hospital, 101 Catharijnesingel, Utrecht, Holland.

from abroad (Canada). But the curves which can be constructed with the figures of Stocks (1959) show that in the metropolitan areas of the U.S.A. no significant retardation of the steeply increasing curve of age specific morbidity is seen between the age of 45 and 55 years, in contrast to the Scandinavian countries (Fig. 2). We shall return to a possible explanation of this fact later.

Several authors have been wondering what this bimodal age distribution actually means. Can we speak of two types of mammary cancer ? And in which respect do these types differ ? Is their cause also different ?

FIG. 1*a*.—Age-specific morbidity (per 100,000 women) of mammary carcinoma in the Netherlands. 3081 cases diagnosed during 1957 and 1958 and collected by the Central Cancer Registry, Amsterdam. It is estimated that about 70 per cent of all cancers are being registered at present ; no correction for this has been attempted. Population figures supplied by the Central Bureau of Statistics, The Hague (population at December 31, 1956).

Maisin and Langerock (1955) made some suggestions as to the nature of the two types. They mentioned *inter alia* that from animal experiments evidence had been obtained that in the genesis of mammary cancer of older age an adrenal dysfunction played a part. Their compatriots Desaive, Lavigne and Adrianne (1958) went a step further at the Perugia Symposium, postulating without any reserve that the type of mammary carcinoma of reproductive life was due to a dysfunction of the ovaries, and the postmenopausal type to a dysfunction of the adrenals.

Ficke and Reiszig (1958) were more cautious, but they too drew attention to the decreasing incidence of the younger type of mammary cancer when the oestrogen production by the ovaries ceases. The curve of the older patients should be the result of a compensating oestrogen production by other endocrine glands. It is known that the adrenals may produce these substances.

Such a hypothesis is not unattractive, for animal experiments have shown that oestrogens—although physiological substances—may under certain conditions have a carcinogenic effect on the mammary gland (Mühlbock and Boot, 1959).

FIG. 1*b*.—Age-specific mortality (per 100,000 women) of mammary carcinoma in the Netherlands. 3191 death certificates registered during 1957 and 1958 by the Central Bureau of Statistics, The Hague. *N.B.* : It is for the greater part about other women than in the graph of morbidity. The anomaly in the curve between 45 and 55 years is less than in the curve of Fig. 1*a* probably due to the longer and inconstant period between onset and registration of the malignancies.

In laboratory animals early bilateral adrenalectomy is equally effective as early castration in lowering the incidence of spontaneous mammary carcinoma (Shimkin and Wyman, 1945). There are many indications that the therapeutic significance of bilateral ovariectomy and adrenalectomy is based on the elimination of oestrogens. If it should be possible to distinguish by clinical or laboratory means between ovarian and adrenal oestrogens the hypothesis could be tested that the two types of mammary cancer may be related to a dysfunction of the ovaries and the adrenals respectively.

Cytological work of Bruinsma and de Waard (1959) seems to enable us to distinguish between these two types. These authors, starting from epidemiological considerations concerning endometrial cancer, wondered whether post-menopausal oestrogen production perhaps would occur in patients with diabetes mellitus, obesity and essential hypertension. A group of 42 diabetics showed indeed a significant higher number of vaginal smears characteristic of oestrogenic activity than did a control group. The difference between these groups was owing to the diabetic patients with obesity and/or hypertension. In the case of

FIG. 2.—Age specific morbidity of mammary carcinoma per year per 100,000 women, after Stocks (1959, Table 4).

patients with obesity and/or hypertension without diabetes oestrogenic smears were frequently encountered too. Because of the limited number of diabetic patients without obesity or hypertension it was impossible to draw any conclusions about a possible difference between these patients and the control group. Of significance were the findings of oestrogenic smears in 17 castrated diabetics. The authors concluded that the oestrogens responsible for these effects were probably of adrenal origin.

De Waard and Baanders (to be published) made a cytological investigation of the urinary sediments of more than 100 women over 55 years of age from the population of Utrecht, Holland, who had been invited to serve as a normal control group in a genetic investigation. There exists a cytological parallelism between the vaginal smear and the urinary sediment (Lencioni, 1953), permitting endocrine evaluation of the latter after staining with Shorr's trichrome stain. The results of this investigation confirmed and completed those of Bruinsma and de Waard (1959) by showing clearly the influence of hypertension and obesity on the frequency of oestrogenic pictures in women with ovaries as well as in those without. Moreover, a study was made of glucose tolerance in the non-obese, non-hypertensive subjects. It was shown that the few women of this subgroup presenting a certain oestrogenic activity had a decreased glucose tolerance. The authors concluded that the combined groups of postmenopausal women with obesity, hypertension and a decreased glucose tolerance contained all those exhibiting oestrogenic activity cytologically. This condition of continuous hormonal activity was called : adrenal oestrus.

### PLAN OF STUDY. MATERIAL AND METHOD

Based on this cytological evidence for adrenal oestrus and on the epidemiological fact of a bimodal age distribution of mammary cancer the following plan of study was made :

Of a number of patients with mammary carcinoma we investigated :

Weight and height.
Blood pressure.
Glucose tolerance.

If obesity, hypertension or a decreased glucose tolerance were present it was assumed that adrenal oestrogen would have been present which could have promoted the carcinogenesis in the mammary gland. If none of these pathological signs was found it was supposed that any carcinogenic influence of oestrogens would have come from the ovaries. Of the " adrenal " as well as of the " ovarian " type of patients with mammary cancer an age distribution could be made ; if these age distributions coincided with the distributions found by epidemiologists in their morbidity statistics an argument would be provided for the correctness of the hypothesis.

The material obtained from 108 patients was collected in six different hospitals. The patients belonged for the greater part to the population of two large towns. Only cases of recent onset (less than six months between first diagnosis and our investigation) in which the malignancy had been confirmed by the pathologist were studied. Cases with distant metastases and those who were or had been treated endocrinologically (with hormone preparations or by surgical intervention) were omitted.

The criteria for classification according to relative weight, blood pressure and glucose tolerance were the following : a body weight more than 25 per cent above Ideal Weight was called obesity ; we used the Table of Ideal Weights given by Bøe, Humerfelt and Wedervang (1957). In laying down the criteria for hypertension we evaluated both the systolic and the diastolic pressure. In the first place the lead given by the Expert Committee on Cardiovascular Disease and Hypertension of the World Health Organisation (1959) was followed, drawing a dividing-line between normal and abnormal blood pressure at 160/95 mm. Hg. This dividing-line has been shown by Morrison and Morris (1959) not to be a purely arbitrary one. However, we considered it justifiable to include as hypertensive cases those with a diastolic pressure of 100 mm. Hg or more if their systolic pressure was at least 150 mm. Hg, and those with a systolic pressure of 170 mm. Hg or more irrespective of diastolic pressure.

The glucose tolerance tests were carried out either pre-operatively or more than 14 days post-operatively in order to avoid the effect of surgical stress. The amount of glucose given was 50 g. by mouth ; the blood sugar estimations were performed in 73 cases according to the method of Hagedorn and Jensen and in 35 according to Folin and Wu.* In judging the blood sugar curves attention was given to both components composing a diabetic type of curve separately : 1. blood glucose level increasing too much, 2. level not decreasing to original (fasting) value within 2 or 2½ hours. Ad 1. we considered the level to be pathological, if at least two values above 180 mg. per cent or one value above 190 mg. per cent was reached. Ad 2. we fixed a dividing line between normal and abnormal after making frequency distributions of the difference between the 2- or 2½-hour values and the fasting values. It was seen that also at higher ages a decrease to below the fasting value often took place, and that a suitable boundary between normal and abnormal was to be found in the region of 2- or 2½-hour levels lying 10–20 mg. per cent above the fasting values. We chose finally as the dividing line : 20 mg. per cent above the fasting value, considering curves with differences of 15–20 mg. per cent dubious ones.

### RESULTS

Based on the above criteria we divided the patients into two groups : one without any pathological feature of the triad—obesity, hypertension or a decreased glucose tolerance, the second with at least one of those features. Three women who were not obese nor hypertensive, but who exhibited a dubious blood sugar curve were classified separately in Table I and omitted from Fig. 3 which thus presents the result of our separation of 105 cases. In this figure which is a graphical condensation of Table I two frequency distributions are seen with modes in the 45–50 and the 60–64 age classes respectively, which show a striking similarity to the distributions expected by epidemiologists. This does not prove that our hypothesis is correct, but it gives definite support to it.

An important question which has to be investigated is : does one in separating any female population according to the presence or the absence of obesity, hypertension and decreased glucose tolerance perhaps find two age distributions similar to the distribution of mammary cancer patients ? It is well known that the features of this triad are much more frequent later in life than in the reproductive period.

We have investigated this point with care, making use of data of a statistical-genetic study designed by two of us (not yet published). The control group needed for this study was obtained by taking 1000 cards of women out of the files of the Population Registry of the town of Utrecht (250,000 inhabitants). This was done at random apart from a certain age distribution. These women were invited to an interview about cancer in their families, together with a brief physical examination including measurement of weight, height, blood pressure and examination of the urine. Almost 60 per cent of these women co-operated ;

* As judged by frequency distributions of the fasting levels the values obtained by the former method are about 5 mg. per cent lower than those obtained by the latter.

FIG. 3.—Separation of 105 cases of mammary carcinoma into two groups (see text).

TABLE I.—*Presence of Obesity, Hypertension and Decreased Glucose Tolerance in 108 Patients with Mammary Carcinoma*

| Age | No pathological features | Obesity only | Hypertension only | Obesity + hypertension | Decreased glucose tolerance only | Obese + decreased glucose tolerance | Hypertension + decreased glucose tolerance | Obesity + hypertension + decreased glucose tolerance | Glucose tolerance dubious | Total |
|---|---|---|---|---|---|---|---|---|---|---|
| 30–34 . | 3 | 1 | — | — | 1 | — | — | — | — | 5 |
| 35–39 . | 1 | 1 | — | — | 1 | 1 | — | — | — | 4 |
| 40–44 . | 5 | 1 | — | — | — | — | — | — | — | 6 |
| 45–49 . | 7 | 2 | 1 | 1 | — | 1 | 1 | 2 | — | 15 |
| 50–54 . | 6 | 1 | 2 | 3 | 3 | 2 | 1 | — | 1 | 19 |
| 55–59 . | 2 | 1 | — | 2 | 3 | 2 | 3 | 1 | 1 | 15 |
| 60–64 . | 1 | 3 | — | 1 | 3 | 2 | 2 | 4 | — | 16 |
| 65–69 . | 1 | 1 | 2 | 1 | — | 2 | 1 | 1 | 1 | 10 |
| 70–74 . | — | 2 | — | — | 2 | 1 | 2 | 3 | — | 10 |
| 75–79 . | — | 1 | — | 2 | 1 | — | — | — | — | 4 |
| 80–84 . | — | — | — | — | 2 | 1 | 1 | — | — | 4 |
| | 26 | 14 | 5 | 10 | 16 | 12 | 11 | 11 | 3 | 108 |

an analysis of their addresses revealed that the living quarters of the higher educational classes were represented slightly better than the other ones. Making a comparison with a group of mammary cancer patients this does not seem to be a draw-back, because it is known that patients with this type of cancer constitute also a slight selection among the higher educational population groups.

The investigation provided us with the data of 571 women. They were divided into two groups according to the same criteria of obesity and hypertension as the 108 patients with mammary cancer (it was impossible to perform a glucose tolerance test in all of them). In view of the shape of the histograms of Fig. 3 we were interested in the question whether obesity and hypertension (occurring singly or combined) were more frequent in the postmenopausal cancer patients than in the postmenopausal controls. Table II shows that this was found indeed. Within both groups there exist small differences between the age classes which are, however, far from significant ($\chi^2$-test). Thus it is permissible to combine the different age classes above 55 years and to summarize as follows : in the mammary cancer group 42 of 59 women are obese and/or hypertensive, that is 71 per cent. In the control group 174 of 322 women have obesity and/or hypertension, that is 54 per cent. Although this latter percentage is high too, the difference from the mammary cancer group is significant (hypergeometrical distribution, $P < 0{\cdot}02$). If the criterion for hypertension is modified in such a way that only blood pressures of at least 170 mm. Hg systolic fall into the pathological group, the difference between the cancer group and the control group becomes even greater ($P < 0{\cdot}01$).

TABLE II.—*Relative Frequency of Obesity and Hypertension (Occurring Singly or Combined) in Mammary Cancer Patients and Controls Over* 55 *Years of Age*

| | | Age | | | | | | |
|---|---|---|---|---|---|---|---|---|
| | | 55–59 | 60–64 | 65–69 | 70–74 | 75–79 | 80–84 | Total |
| Mammary cancer group | Number of women | 15 | 16 | 10 | 10 | 4 | 4 | 59 |
| | Number of women with obesity and/or hypertension | 9 | 12 | 8 | 8 | 3 | 2 | 42 |
| Control group | Number of women | 78 | 77 | 67 | 45 | 36 | 19 | 322 |
| | Number of women with obesity and/or hypertension | 40 | 45 | 38 | 21 | 18 | 12 | 174 |

Thus the conclusion seems to be justified that mammary carcinoma after the menopause has a preference for the obese and the hypertensive, occurring almost exclusively in women with obesity and/or hypertension and/or a decreased glucose tolerance, conditions which are associated with oestrogenic activity of adrenal origin.

### DISCUSSION

The direct evidence of a connection between adrenal oestrogen production and a decreased glucose tolerance in older people not yet being amply present, some indirect evidence may be wanted.

It is known that older patients with diabetes mellitus with or without obesity who do well on a dietary regime alone or in conjunction with oral administration of sulfonylurea derivatives (like tolbutamide, etc.) produce insulin in reasonable amounts ; their diabetes is considered a " Gegenregulationsdiabetes " (Bertram, Bendfeldt and Otto, 1956). Those who are of the opinion that the adrenals play a part in this " Gegenregulation " (Bastenie, 1956) find support in the observations of Szenas and Pattee (1959) that the glucocorticoid production in obesity is increased. This influence of overweight is also to be gathered from the statistical data of Borth, Linder and Riondel (1957). Moreover the decreased glucose tolerance which is so often found in obese subjects is of the G.I.T.T.-positive type, pointing to increased secretion of glucocorticoids too (Arendt and Pattee, 1956).

There exists a certain parallelism between the production of glucocorticoids and adrenal oestrogens : operative stress or injection of ACTH increases the urinary excretion of both types of steroids (Décourt *et al.*, 1951 ; Bulbrook *et al.*, 1958 ; Brown, Falconer and Strong, 1959) and cortisone therapy inhibits the production not only of glucocorticoids but also of adrenal oestrogens (Smith and Emerson, 1954 ; Block, McCarthy and Vial, 1959*b*).

We are not of the opinion that every curve revealing decreased glucose tolerance is of necessity a reflection of an increased secretion of glucocorticoids (it would have been preferable if also glucose-insulin tolerance tests of our patients could have been made). However, it seems reasonable to assume with French gerontologists that at older age in the larger part of these curves the adrenal cortex plays a part (Binet and Boulière, 1955), and such an assumption already satisfies the epidemiologist.

If our observations can be confirmed by others, a definite perspective regarding mammary cancer is looming up. In the first place the indications in the treatment of metastasized mammary carcinoma could get a more theoretical basis. In judging the results of different hormonal kinds of therapy the type of cancer (" ovarian " or " adrenal ") could be included in the considerations. Our curve of the " ovarian " type (Fig. 3) fits well in with the observations of Dao and Huggins (1957) that after 54 years of age bilateral oöphorectomy is seldom indicated. It could be found that young women with obesity and/or hypertension are not helped sufficiently by castration alone but that in them at an early stage the elimination of adrenal oestrogens must be advocated, either by surgical means or by suppression of pituitary ACTH with corticosteroids. Finally the fact could be understood why some patients who did not react favourably to surgical castration were subsequently greatly helped by adrenalectomy (Block *et al.*, 1959*a*), the poor castration response not being based on hormone independence but on the fact that the ovaries were inactive in contrast to the adrenals.

But of no less significance seems to us the possibility for more insight into some aetiological factors of mammary carcinoma. We venture to present the following hypothesis :

It is well known that heredity and environment play a part in the genesis of mammary cancer. Concerning the environmental factor(s) it must be stipulated that this carcinoma has a preference for the higher social classes (Stocks, 1955) and that it is seen more frequently in Western countries than in peoples of Asia and Africa (Segi *et al.*, 1957 ; Oettlé and Higginson, 1958).

It is also well known that the disease entities of the triad obesity, essential hypertension and diabetes mellitus (" diabète gras ") are more frequent in the materially blessed peoples of Western countries than in these African and Asian peoples (de Langen, 1958 ; Smirk, 1949). Further, obesity, hypertension and diabetes have also a hereditary aspect.

We suppose that the phenomenon of adrenal oestrus on account of its association with obesity, hypertension and decreased glucose tolerance (not only in statistical but probably also in pathophysiological respect) has also a hereditary and an environmental aspect, and that these aspects in turn determine the hereditary and the environmental aspects of the " adrenal " type of mammary carcinoma.

With this hypothesis the peculiar feature shown in Fig. 2 could be explained why the bimodality of the age-specific frequency curve of mammary cancer is not pronounced in the metropolitan areas of the U.S.A. in contrast to the Scandinavian countries. In these parts of the U.S.A. the Western technical civilization finds its summit, with all the life habits (nutrition *inter alia*) inherent to it. If diabetes, obesity and hypertension and the phenomenon of adrenal oestrus begin to occur a few years earlier in life and if their frequencies increase with age somewhat stronger than elsewhere in the Western world, the " adrenal " group of mammary cancers will overlap the " ovarian " group almost completely masking the decrease of the latter group at menopausal age.

This environmental aspect of the older type of breast cancer may also explain certain facts mentioned by McMahon (1957), himself a critic of the bimodal type hypothesis, namely, (1) the difference in mortality trends during the 20th century between mammary cancer among pre- and postmenopausal women respectively, (2) the shift in the incidence break of the age specific morbidity curves of breast cancer in Connecticut, (3) the differences between the curves of Danish urban and rural areas.

In contrast to the life habits of Western countries we know of peoples with ways of living in which diabetes, obesity and hypertension are relatively unknown. Perhaps by changing our nutritional habits we might be able to reduce not only the incidence of these diseases but also that of adrenal oestrus and of the " adrenal" type of mammary carcinoma.

### SUMMARY

Morbidity and mortality statistics suggest that the population of mammary cancer patients in Western countries is composed of two populations, each with its own age distribution having their highest frequencies about 48 years and 65 years of age respectively. Statistics from the Netherlands show this bimodal distribution too.

In trying to find a possible basis for this phenomenon the hypothesis is discussed that the type of mammary cancer of reproductive age is caused by an ovarian dysfunction and the type of older age by an adrenal dysfunction. This hypothesis is tested by applying epidemiologically the fact established by endocrine cytology that patients with obesity, essential hypertension and/or a decreased glucose tolerance often show signs of oestrogenic activity which are very probably of adrenal origin.

The results of this application seem to be in agreement with the mentioned hypothesis. The possible significance of these facts for more insight into the hereditary and the environmental aspects of mammary cancer is discussed.

The authors wish to thank those colleagues who helped to collect the material for study : H. J. H. Bolhuis ; K. Breur and O. Karpiak-van Driel (Radiotherapeutisch Instituut, Rotterdam) ; Prof. Dr. J. F. Nuboer (Heelkundige Universiteitskliniek, Utrecht) ; D. Miete (Gemeente Ziekenhuis, Arnhem) ; J. H. Lichtenbelt ; A. L. E. Schaepkens van Riempst (Antonius Ziekenhuis, Utrecht) ; A. Stofberg (Geertruiden Ziekenhuis, Deventer) and Dr. E. Verschuyl. The cancer morbidity figures were supplied by L. Meinsma, director of the Central Cancer Registry. Dr. E. Tonkes and Miss N. Vermeulen gave their valuable assistance in the investigation of the control group.

### REFERENCES

Anderson, E., Reed, S. C., Huseby, R. A. and Oliver, C. P.—(1950) *Cancer*, **3**, 410.

Arendt, E. C. and Pattee, C. J.—(1956) *J. clin. Endocrin.*, **16**, 367.

Basténie, P.—(1956) ‘Cortico-surrénale et diabète humain’. Paris (Masson).

Bertram, F. , Bendfeldt, E. and Otto, H.—(1956) *Dtsch. med. Wschr.*, **81**, 274.

Binet, L. and Bourlière, F.—(1955) ‘Précis de Gérontologie’. Paris (Masson), p. 479.

Block, G. E., Burgess Vial, A., McCarthy, J. D., Porter, C. L. W. and Coller, F. A.—(1959*a*) *Surg. Gynec. Obstet.*, **108**, 651.

*Idem*, McCarthy, J. D. and Burgess Vial, A.—(1959*b*) *Arch. Surg.*, **78**, 732.

Bøe, J., Humerfelt, S. and Wedervang, F.—(1957) *Acta med. scand.*, Suppl. 321, Table 50, p. 145.

Borth, R., Linder, A. and Riondel, A.—(1957) *Acta endocr., Copenhagen*, **25**, 33.

Brown, J. B., Falconer, C. W. A. and Strong, J. A.—(1959) *J. Endocrin.*, **19**, 52.

Bruinsma, A. H. and de Waard, F.—(1959) *Acta endocr., Copenhagen*, **32**, 233.

Bulbrook, R. D., Greenwood, F. C., Hadfield, G. J. and Scowen, E. F.—(1958) *Brit. med. J.*, ii, 7.

Clemmesen, J.—(1948) *Brit. J. Radiol.*, **21**, 583.

Dao, T. L. and Huggins, C.—(1957) *J. Amer. med. Ass.*, **165**, 1793.

Décourt, J., Jayle, M. F., Crépy, O. and Judas, O.—(1951) *Ann. d'Endocr.*, **12**, 719.

Denoix, P. F.—(1958) *Proc. 2nd int. Symp. on Mammary Cancer, Perugia*, p. 285.

Desaive, P., Lavigne, J. and Adrianne, A.—(1958) *Ibid.*, p. 37.

Ficke, K. H. and Reiszig, G.—(1958) *Arch. Geschwulstforsch.*, **12**, 379.

Jacobsen, O.—(1946) ‘Heredity in breast cancer’. London (Lewis & Co.).

de Langen, C. D.—(1958) *Geneesk. Bl.*, **48**, No. 3.

Lencioni, L. J.—(1953) *J. clin. Endocrin.*, **13**, 263.

McMahon, B.—(1957) *Cancer*, **10**, 1037.

Maisin, J. H. and Langerock, G.—(1955) *Schweiz. Z. Path.*, **18**, 690.

Morrison, S. L. and Morris, J. N.—(1959) *Lancet*, ii, 864.

Mühlbock, O. and Boot, L. M.—(1959) Ciba Foundation Symposium on Carcinogenesis. London (Churchill), p. 83.

Oettlé, A. G. and Higginson, J.—(1958) *Proc. 2nd int. Symp. on Mammary Cancer, Perugia*, p. 203.

Pedersen, E. and Magnus, K.—(1959) Cancer Registration in Norway, Monograph No. 1 of the Norwegian Cancer Society.

Phillips, A. J. and Owchar, M.—(1957) *Bull. World Hlth Org.*, **16**, 267.

von Pirquet, C.—(1930) ' Allergie des Lebensalters: die bösartigen Geschwülste'. Leipzig (Thieme Verlag).
Segi, M., Fukushima, I., Fujisaku, S., Kuzihara, M., Saito, S., Asano, K., Nagaika, H., Noye, Y. and Kamoi, M.—(1957) *J. nat. Cancer Inst.*, **18**, 373.
Shimkin, M. B. and Wyman, R. S.—(1945) *Ibid.*, **6**, 187.
Smith, O. W. and Emerson, K.—(1954) *Proc. Soc. exp. Biol. N.Y.*, **85**, 264.
Smirk, F. H.—(1949) *Brit. med. J.*, i, 791.
Stocks, P.—(1955) *Schweiz. Z. Path.*, **18**, 706.—(1959) *Bull. World. Hlth Org.*, **20**, 697.
Szenas, P. and Pattee, C. J.—(1959) *J. clin. Endocrin.*, **19**, 344.
de Waard, F. and Baanders, E. A.—To be published in *Acta Endocr., Copenhagen.*
World Health Organisation.—(1959) *Tech. Rep. Ser.* No. 168.